# The SHID wound classification system for diabetic foot ulcer patients: a validity study

**DOI:** 10.25122/jml-2022-0090

**Published:** 2022-10

**Authors:** Suriadi Jais, Kharisma Pratama, Jery Fahrain, Junaidi Junaidi, Tutur Kardiatun, Uji Kawuryan

**Affiliations:** 1 Department of Medical Surgical Nursing, Sekolah Tinggi Ilmu Keperawatan Muhammadiyah Pontianak, Pontianak, Indonesia

**Keywords:** SHID, wound classification, predictive validity, ABI – ankle brachial index, AUC – area under the curve, CI – confidence interval, DFU – diabetic foot ulcer, PEDIS – Perfusion, Extent, Depth, Infection, and Sensation, RN – registered nurse, ROC – receiver operating characteristic, SHID – Suriadi, Haryanto, Imran, Defa, SINBAD – Site, Ischemia, Neuropathy, Bacterial Infection and Depth, TU – Texas University

## Abstract

This study aimed to evaluate the predictive validity of the SHID (Suriadi, Haryanto, Imran dan Defa) wound classification system compared to TU (Texas University) and Wagner wound classification systems in Indonesia. A prospective cohort study included patients with diabetic foot ulcers at Kitamura wound clinic in Indonesia. A total of 111 diabetic foot ulcer patients were assessed with SHID, TU, and Wagner wound classification systems. Two postgraduate nursing students assessed 111 wounds of bedside patients and observed them for 4 weeks. The predictive validity test indicated that the cut-off score of ≤grade 2 for SHID was 74% and 97%, ≤grade IB for TU was 77% and 92%, then ≤grade 2 for Wagner was 84% and 71% for sensitivity and specificity, respectively. The area under the curve (AUC) in SHID, TU, and Wagner tools was 0.90 (95% CI: 0.828–0.950), 0.85 (95% CI: I0.766–0.910), and 0.86 (95% CI: 0.775–0.917), respectively. The Youden index for SHID, TU, and Wagner was 0.72%, 0.70%, and 0.55%, respectively. The wound classification systems are good tools for identifying diabetic foot ulcers. However, the newly developed SHID tool produced the best AUC and Youden Index values compared to the Wagner tool.

## INTRODUCTION

Diabetes is a chronic health problem with serious complications, such as diabetic foot ulcers. The global prevalence of diabetic foot ulcers (DFU) affecting wound onset and healing is 6.3%, and 5.5% in Asia [1]. Furthermore, patients with diabetic foot ulcers have serious health problems and major challenges due to infections or neurovascular complications. These patients may undergo amputations, which have a negative impact on their quality of life and survival [2]. An infection of diabetic foot ulcers is a common clinical problem, where about 50% of patients become infected, require amputation, and die within 5 years [3]. Therefore, this poses a heavy burden on the government, healthcare providers, and society, although these wound problems are probably caused by an inaccurate assessment and/or diagnostic approach [4]. Accurate wound assessment is essential to ensure appropriate patient care and wound management.

Identifying diabetic foot ulcers and their prognostic risks at the beginning of the assessment and accurate prediction of abnormal condition are necessary to prevent further complications and negative outcomes. Subsequently, the prognosis of diabetic foot ulcers is assessed using the Wagner, Texas University (TU), Site, Ischemia, Neuropathy, Bacterial Infection And Depth (SINBAD), and Perfusion, Extent, Depth, Infection, And Sensation Scale (PEDIS) wound classification systems [5]. Although the tools have been studied for validity, the systems are still difficult to use in clinical practice due to the complexity of the grading system employed and are perhaps better suited for research purposes, such as PEDIS [5–9]. The Wagner classification system was the early framework for classifying diabetic foot ulcers. It evaluates the depth of the ulcer and the existence of osteomyelitis or gangrene and divides the ulcers into six grades [10]. Although it is simple to apply and in a wide application, it does not recognize peripheral arterial disease and infection for the first three grades (0–2) [11].

Meanwhile, the TU system comprises grades and stages. Grades of diabetic foot ulcers are based on ulcer depth, and the stages are completed by the presence or absence of infection and ischemia. The TU classification system was a good predictor of outcome [12]. However, there is controversy over validating Wagner and Texas University classification systems [10–14]. So far, neither has been universally accepted as a standardized method for DFU assessment [10].

SHID (Suriadi, Haryanto, Imran, and Defa) is a wound classification system developed in Indonesia [6] since the existing classification is difficult for nurses to use and due to the considerations of varying reliability, validity, and consistency of other wound classification systems [2, 8, 15, 16]. Assessment instruments must be both reliable and valid for study results to be credible [17]. Hence, a reliability study on two widely used wound classification system tools, such as Wagner and TU, and a newly developed SHID classification have been conducted in Indonesia (Table 1). The results of the inter-rater reliability study between expert nurses on the three wound classification showed almost perfect agreement for SHID scale (Kappa=0.81; 95% CI 0.65–0.97), substantial for Wagner scale (Kappa=0.77; 95% CI 0.52–0.96), and moderate for University of Texas scale (Kappa=0.50; 95% CI 0.09–0.90) [6]. The purpose of this study was to evaluate the predictive validity of SHID compared to Wagner and TU wound classification systems.

**Table 1:** SHID wound classification.

Ulcer grading	Description
1	Epidermis and/or to the dermis
2	Epidermis and/or dermis with any one or more signs of infection/ischemic/osteomyelitis (X-ray)
3	Subcutaneous/fascia/muscle/tendon
4	Subcutaneous/fascia/muscle/tendon with any one or more signs of infection/ischemia/osteomyelitis (with x-ray)
5	Subcutaneous/fascia/muscle/tendon/joint-capsule/bone
6	Subcutaneous/fascia/muscle/tendon/joint- capsule/bone/with any one or more signs of infection/ischemia/osteomyelitis (with x-ray)

## Material and Methods

### Research design

A prospective cohort design was conducted between April 2020 and September 2021. This was performed to evaluate the predictive validity of three wound classification systems, including SHID, Wagner, and TU.

### Participants

Applying the inclusion criteria, we included patients who entered the Kitamura wound clinic with type II diabetes and foot wounds, both new and recurrent and outpatients. Also, the patients did not experience moderate to severe complications, were cooperative, gave informed consent, and were from Indonesia. Subsequently, the patients could be withdrawn from the study at any time without any reason.

### Sample and data collection

The purposive sampling method was used to collect the sample, where the patients were selected based on the objectives of the study. This study involved two postgraduate students trained on how to assess and use the three wound classification systems for diabetic foot ulcer patients. Patients were first assessed at admission with three wound classification assessment tools. Research assistants conducted assessments and examinations on patients independently, with the two assistants not knowing each other when performing wound assessments using these three tools. In this investigation, the patients were evaluated and followed up for 4 weeks for healing and/or discharged [18–20]. Complete healing was defined as ulcer closure with no need for any dressing [21]. Wound care was carried out by a diabetic wound nurse on duty at the time.

### Instruments

This study used several devices for data collection, including vascular Doppler to examine the ABI (ankle-brachial index), monofilament test to examine neuropathy, demographic data assessment, and wound classification tools. Also, the HbA1C samples were taken.

### Data analysis

Patients' characteristics and wound status were evaluated using descriptive and univariate analysis. The assessment of wound grading data from three wound classification tools was then analyzed by receiver operating characteristic (ROC) to evaluate the accuracy and diagnostic probabilities of the tools, such as sensitivity and specificity. Sensitivity is the proportion of patients with the disorder who have a positive test, including the percentage of wound healing in 4 weeks, with scores more or equal to the cut-off. Meanwhile, specificity is the proportion of patients without the disorder with a negative test comprising the percentage of wound healing in more than 4 weeks, with scores more or equal to the cut-off. The ROC is a graphical representation of sensitivity (true-positives) on the y-axis *versus* specificity (false-positives) on the x-axis over the possible cut-off scores. Therefore, the ROC provides a measure of the trade-off between the true positive rate *versus* the false positive rate over the possible dichotomous cut-off scores for a test. The overall validity of the wound classification systems was assessed by calculating the area under the curve (AUC) of the ROC, with a higher AUC arising from more accurate tests [22]. The data was calculated using MedCalc^®^ Belgium, version 15.8.

## Results

### Characteristics of participants and wounds

About 111 patients were eligible, with 31 healing wounds in 4 weeks and 80 non-healing wounds used to determine the predictive validity of the SHID, Wagner, and TU wound classification systems. Table 2 shows the patients' characteristics. The median age was 56 (32–82) years; 61 patients (60.4%) were female; 52 patients (46.8%) had neuropathy; the median duration of diabetes was 5 (0–26) years; 94 patients (84.7%) were smoking; median HbA1C was 11.2% (6 –14); median ABI was 1 (0.6–1.8); median BMI (body mass index) was 21.9 (15.5–36.2). Table 3 illustrates the characteristics of wounds, where 57 patients (51.4) had recurrent wounds, 57 patients (51.4) had wound locations at the forefoot, and 61 wounds (55.0%) were triggered by trauma. Furthermore, the causes of trauma leading to wounds in diabetic patients include a needle, fishbone, shoes/sandal, nail puncture, bite from an insect, scratching, glass injury, and fall.

**Table 2:** Characteristics of participants.

Characteristics	
Age (median years) n=111	56 (32–82)
Gender no (%) n=111	
**Male**	67 (60.4)
**Female**	44 (39.6)
Neuropathic no (%) n=111	
**Positive**	52 (46.8%)
**Negative**	59 (53.2%)
Smoking n=111	
**Smoking**	7 (6.3%)
**No smoking**	94 (84.7%)
BMI (median kg/m^2^) n=111	21.9 (15.5–36.2)
Duration of DM (median years) n=111	5 (0–26)
HbA1C (median %) n=82	11.2 (6–14)
ABI (median) n=111	1 (0.6–1.8)

**Table 3:** Characteristics of wounds.

Characteristic	
Wound status no (%)	
**New**	54 (48.6)
**Recurrence**	57 (51.4)
Wound healing in 4 weeks no (%)	
**Healing**	31 (27.93)
**Non healing**	80 (72.07)
Wound location no (%)	
**Forefoot**	57 (51.4)
**Midfoot**	36 (32.4)
**Hindfoot**	18 (16.2)
Trigger no (%)	
**Trauma**	61 (55.0)
**Diabetic bullae**	21 (18.9)
**Unknown**	29 (26.1)

### Predictive validity

**Figure 1:**
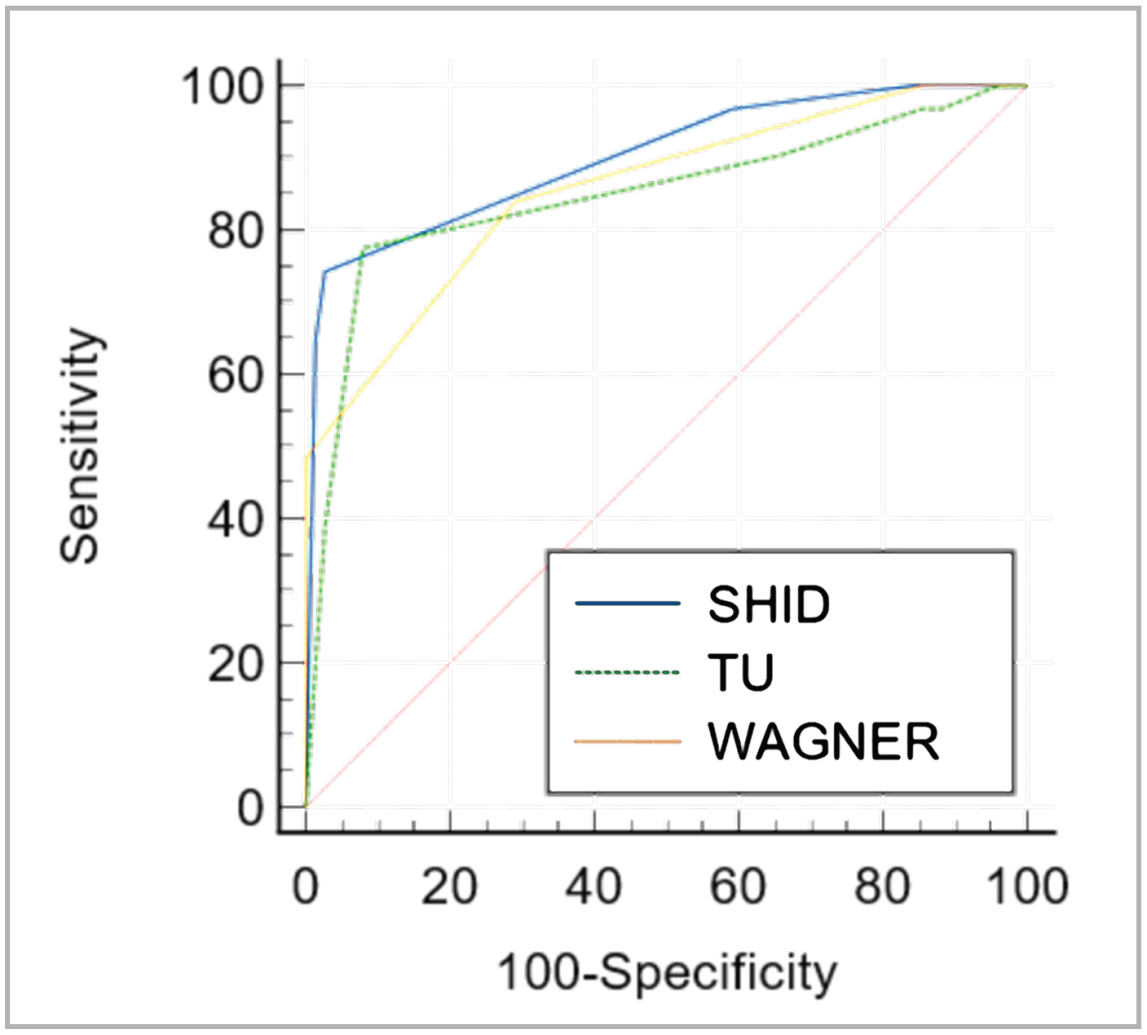
The receiver operator characteristic curve of the SHID, TU, and Wagner wound classification for wound healing within 4 weeks of follow-up (n=111). Area under curve for SHID, TU, and Wagner wound classifications was 0.901 (95% CI, 0.828–0.950), 0.848, (95% CI: I0.766–0.910) and 0.856 (95% CI, 0.775–0.917), respectively.

The predictive validity study demonstrated that the wound classification systems produced a good sensitivity and specificity of 77% and 92% with cut-off scores ≤grade IB for TU, 74% and 97% with cut-off scores ≤grade 2 for SHID, and 84% and 71% with cut-off scores ≤grade 2 for Wagner, respectively (Table 4). In Figure 1, sensitivity was plotted against specificity for each possible score of the SHID wound classification to generate the ROC, and the AUC was 0.901 (95% CI: 0.828–0.950). However, TU and Wagner had an AUC of 0.848 (95% CI: I0.766–0.910) and 0.856 (95% CI: 0.775–0.917), respectively. The Youden index for SHID, TU, and Wagner was 0.715%, 0.695%, and 0.549%, respectively (Table 4).

**Table 4:** Sensitivity and specificity of SHID, TU, and Wagner wound classification.

Wound classification	Cut-off scores	Sensitivity	Specificity	Youden index
SHID	≤2	74.19	97.37	0.7156
TU	≤IB	77.42	92.11	0.6952
Wagner	≤2	83.87	71.05	0.5492.

## Discussion

This study showed that the three wound classification systems were valid assessment tools for evaluating wounds in diabetic patients. Our study presented that the SHID, TU, and Wagner correctly identified 74%, 77%, and 84% of the patients with wound healing in 4 weeks, respectively, although they failed to identify the remaining 26%, 23%, and 16%, respectively. However, SHID, TU, and Wagner correctly identified 97%, 92%, and 71% of the patients without wound healing in 4 weeks, respectively, and also identified 3%, 8%, and 29% of patients as having wound healing when they do not, respectively. Specificity refers to the percentage of patients that do not heal within 4 weeks and are tested as negative. Therefore, diagnostic tests with high specificity have few false-positive results, while those who tested positive were mostly positive [23]. The comparison of the predictive validity of 3 wound classification systems showed that the SHID and TU had better predictive validity, based on Youden's index (72% for SHID, 70% for TU, and 55% for Wagner). Furthermore, the Youden index of the Wagner system was lower than others, probably due to inadequate ischemia and infection handling [24]. This system is also limited as a surgical tool to identify and describe vascular disease as an independent risk factor for poor outcomes. In addition, superficial wounds that are infected or have a vascular component without gangrene cannot be classified separately [24]. The SHID wound classification is a new model developed specifically to assess DFU classification. Other wound classification systems, such as the SINBAD system, have entered items on neuropathy. The SHID system does not consider that neuropathy can arise at all wound classification levels, from grades one to six. Neuropathy can also develop in patients who have no ulcers, which may complicate the identification of wounds by practitioners when included.

Although these three wound classifications specific to diabetic patients are valid tools, the SHID showed excellent results in the AUC scores compared to TU and Wagner. Hence, this indicates that SHID can be used to estimate the discriminative power of a test. The closer the curve to the upper-left-hand corner and the larger the AUC, the better the test is at discerning between wound healing time within and outside four weeks.

This study presented that SHID, TU, and Wagner systems predict that DFU wounds heal within 4 weeks. Also, it concluded that the healing achieved in approximately 4 weeks is probably due to the degree of superficial wound, the standard of care applied, and if there are no problems with the pathology [25]. However, this study differs slightly from a previous study regarding the Wagner system, which reported that a 5-week median wound healing rate was achieved in grades 1–2 and/or superficial wounds. Also, TU reported that the degree of a wound with low severity (grade 1, stage A/B or grade 2, stage A) had a wound healing time of 2 weeks [25]. This is possibly due to various factors, such as the wound conditions, patient variables, clinical settings, standard of wound care, and clinician experience [26–28]. One study reported that TU and Wagner on wound dept had sensitivity and specificity values of 75% and 94%, and 73% and 96%, respectively. They showed substantial accuracy, and their main variables were associated with lower extremity amputation occurrence [21]. However, SHID has not evaluated the validity of the wound dept; hence, it cannot be compared.

Our study found that the superficial grade of wound classification based on SHID, TU, and Wagner systems had no lower extremity amputations compared to previous studies, which reported 4–8% in Wagner and 3% in TU systems [10, 11], because of the pathological circumstances [26]. In addition, the Wagner system is favorable in predicting the degree of deep or grade 3 and above wound with the incidence of amputation and wound healing in the long term [11, 21, 29, 30]. Although the TU system was used in many clinical trials and diabetic foot centers [5, 11], it is difficult to use in clinical settings in Indonesia except for research purposes [6, 31]. This is confirmed by a previous inter-reliability study, where the inter-reliability values on the Kappa statistic were high for expert nurses (0.63) compared to registered nurses (0.34), and this is similar to the Wagner system, which showed 0.43 and 0.77 for registered nurses (RN) and expert nurses, respectively [6]. Meanwhile, SHID produced excellent scores for RN (0.81) and perfect scores for expert nurses (1.00); hence, it can be considered when using the TU and Wagner wound classifications in Indonesia. However, further studies are necessary with multiple observers and various settings to determine the reliability and validity of SHID wound classification and respective outcomes. This study postulated that SHID is a tool with a cut-off score ≤grade 2, which can be useful in the healthcare setting, especially in identifying the early diagnosis of diabetic ulcers and their prognosis in healing within 4 weeks by a nurse. The SHID wound classification system is new and has shown excellent predictive validity. Therefore, it can be used for the early identification of diabetic wounds and estimation of their prognosis in clinical settings. However, nurses need to be trained to use this tool. The data set used in this study was obtained from one wound clinic; hence, limiting its generalizability. Therefore, further validation studies should be carried out on larger samples and in different settings with a longer follow-up.

## Conclusion

This study demonstrated that SHID wound classification had the best predictive validity values, followed by TU wound classification. SHID wound classification identifies early wound diagnoses in diabetic patients; hence, it can be beneficial in clinical settings. Therefore, SHID wound classification systems should be evaluated in a clinical setting and/or hospital. Although the results showed that SHID wound classification had good predictive validity, future studies are necessary to evaluate the cut-off value in the long term and the outcomes in other clinical environments.

## Data Availability

The data that support the findings of this study are available from the corresponding author upon reasonable request.

## Personal thanks

The authors thank the participants and colleagues who contributed to this study.
